# A comparative analysis of telomere length maintenance circuits in fission and budding yeast

**DOI:** 10.3389/fgene.2022.1033113

**Published:** 2022-11-04

**Authors:** Iftah Peretz, Martin Kupiec, Roded Sharan

**Affiliations:** ^1^ The Shmunis School of Biomedicine and Cancer Research, Tel Aviv University, Tel Aviv, Israel; ^2^ Blavatnik School of Computer Science, Tel Aviv University, Tel Aviv, Israel

**Keywords:** machine learning model (ML model), pathways, protein complexes, telomere length maintenance (TLM), genetic interactions, *Saccharomyces cerevisiae* (Baker’s yeast), *Schizosaccharomyces pombe* (fission yeast)

## Abstract

The natural ends of the linear eukaryotic chromosomes are protected by telomeres, which also play an important role in aging and cancer development. Telomere length varies between species, but it is strictly controlled in all organisms. The process of Telomere Length Maintenance (TLM) involves many pathways, protein complexes and interactions that were first discovered in budding and fission yeast model organisms (*Saccharomyces cerevisiae*, *Schizosaccharomyces pombe*). In particular, large-scale systematic genetic screens in budding yeast uncovered a network of ≈ 500 genes that, when mutated, cause telomeres to lengthen or to shorten. In contrast, the TLM network in fission yeast remains largely unknown and systematic data is still lacking. In this work we try to close this gap and develop a unified interpretable machine learning framework for TLM gene discovery and phenotype prediction in both species. We demonstrate the utility of our framework in pinpointing the pathways by which TLM homeostasis is maintained and predicting novel TLM genes in fission yeast. The results of this study could be used for better understanding of telomere biology and serve as a step towards the adaptation of computational methods based on telomeric data for human prognosis.

## 1 Introduction

In most eukaryotes, the chromosomal ends are protected by telomeres, composed of short G-rich repeats and a special set of proteins (Blackburn, 1991). Telomeres play a pivotal role in chromosomal duplication, stability, and dynamics (Zakian, 1995).

Telomeres shrink with replicative age due to the inability of DNA polymerases to synthesize lagging-strand DNA after the removal of RNA primers at the extreme ends of the chromosome (Hayflick, 1965; Harley et al., 1994). This condition, referred to as the “end replication problem”, is solved by the ribonucleoprotein telomerase, which uses its RNA subunit to reverse transcribe telomeric DNA (Greider and Blackburn, 1989). Telomerase is expressed in stem cells, but is barely detected in somatic cells. Continuously growing microorganisms, such as yeasts, constitutively express telomerase and are excellent models to investigate the mechanisms that regulate telomere biology and have uncovered a complex network of factors required to maintain telomere length homeostasis (Wellinger and Zakian, 2012; Harari and Kupiec, 2014; Kupiec, 2014). In budding yeast (*S. cerevisiae*), telomerase recruitment and activity is mediated by several factors that are required for the elongation of the shortest telomeres in some of the cell cycles (Teixeira et al., 2004).

Large-scale systematic genetic screens in *S. cerevisiae*, which scored collections of gene-knockouts and hypomorphic alleles, discovered a network of genes that participate in controlling telomere length (Askree et al., 2004; Gatbonton et al., 2006; Ungar et al., 2009; Puddu et al., 2019). These Telomere Length Maintenance (TLM) gene products have a variety of biochemical roles, some of which were not previously identified to be connected with the regulation of telomere size. Whereas mutations in some of these genes lead to shorter telomeres, others cause telomeres to elongate. Thus, each and every one of the ≈ 500 genes identified controls in a positive or negative way the length of telomeres.

So far, to the extent that we know, no attention has been paid to computationally modeling the TLM network in *S. pombe*. Thus, the aim of this study is to close this gap and to create a machine learning framework for examining telomere maintenance in fission and budding yeast. Our proposed framework is first validated in *S. cerevisiae* for detecting telomeric length phenotype. Next, we test it on curated *S. pombe* TLM data, and pinpoint the most important pathways and protein complexes that make up TLM homeostasis. We follow by investigating the TLM phenotype within the yeast orthologs and suggest the most likely *S. pombe* genes that are currently unknown to be members of the TLM network in this organism. Last, we perform gene ontology (GO) enrichment analysis for these candidates and reveal that they are significantly enriched for biological processes known to be highly linked with telomere maintenance functions.

## 2 Materials and methods

### 2.1 Telomere length data

The data for the *S. cerevisiae* TLM genes along with their corresponding telomere length category was obtained from Van Leeuwen et al. (2016) and Puddu et al. (2019). For the binary classification of telomere length, the categories were reduced to the following phenotypes: ‘short’ and ‘long’. *YIR016W* was observed as both normal and ‘very long’, hence we assigned it as ‘long’. The data also included the Decreased Abundance by mRNA Perturbation (DAmP) collection and we treated the two telomere length labels of ‘DAmP Short’ and ‘DAmP Long’ as ‘short’ and ‘long’, respectively.

The *S. pombe* TLM genes were curated from the Fission Yeast Phenotype Ontology (FYPO) v2011-01-18 (Harris et al., 2013). The following FYPO terms were labeled as having ‘short’ ('FYPO:0002239′, 'FYPO:0006511′, 'FYPO:0003106′, 'FYPO:0003107′) and ‘long’ ('FYPO:0002019′) telomere length phenotypes. Finally, we merged these genes with a list of genes that were found to regulate the homeostasis of telomeres (Liu et al., 2010).

Overall, we obtained 483 and 224 unique TLM genes with corresponding binary telomere length phenotypes for *S. cerevisiae* and *S. pombe*, respectively (Supplementary Table S1).

### 2.2 Genetic interaction data

For each *S. cerevisiae* TLM gene, raw Genetic Interaction (GI) scores were taken from the pairwise interaction format of the TheCellMap.org repository (Usaj et al., 2017). They were produced by systematic Synthetic Genetic Array (SGA) experiments and scored by comparing the fitness of the double mutant to the corresponding single mutants (Costanzo et al., 2016). We only considered the authors’ lenient threshold, i.e., GIs with *p* − *value* < 0.05. In the case of multiple measurements per interaction, the GI score with the lowest *p* − *value* was used. The outcome was a score matrix of the TLM genes and 5850 genes sharing at least one GI with one of the TLM genes.

The *S. pombe* GI data was downloaded from BioGRID v4.4.207 (Oughtred et al., 2021). Unlike the *S. cerevisiae* data, the interactions only contain a verbal description and not a numerical score, which we mapped into a score of 1 when there was an interaction and 0 otherwise. We used all the data that are marked ‘genetic’ in their ‘Experimental System Type’ field.

### 2.3 Pathway data

To construct the pathway features, the Kyoto Encyclopedia of Genes and Genomes (KEGG) pathway database release 99.0 (Ogata et al., 1999) was parsed *via* the BioServices v1.10.1 (Cokelaer et al., 2013) API. Only non-global and non-overview maps were considered (i.e., utilizing pathways that only contain genes and not ones that contain other pathways).

### 2.4 Protein complex data

For *S. cerevisiae*, the CYC2008 catalog was used. It contains 408 manually curated heteromeric protein complexes that were confirmed by small-scale experiments from the literature (Pu et al., 2009).

*S. pombe* complex information was downloaded from PomBase (Harris et al., 2022). It is based on the GO database, for terms that are classified under “macromolecular complex” (GO:0032991) and have fission yeast genes annotated, such that the most specific complex is retained.

### 2.5 Orthology data

A manually curated ortholog list of fission to budding yeast was retrieved from PomBase (Wood et al., 2012). For cases where there was more than one ortholog per gene, the gene with the maximum score from the Smith-Waterman alignment algorithm (Smith and Waterman, 1981) was selected. This was achieved using the Biopython v1.79 (Cock et al., 2009) pairwise2.align.localds function with the same parameters as in the web BLAST NCBI interface (http://blast.ncbi.nlm.nih.gov), i.e., BLOSUM62 scoring matrix, a gap cost of 11 and an extension cost of 1. The final set contained 3953 orthologous pairs (Supplementary Table S2).

### 2.6 GO data

In order to process the GO consortium database (Ashburner et al., 2000; Consortium, 2021) the Python package GOATOOLS v1.2.3 (Klopfenstein et al., 2018) was used. For the feature engineering, GO terms from Biological Process (BP) and Cellular Component (CC) categories were filtered to include only genes for which we have prior data (i.e., genes that appear in the *S. cerevisiae* GI dataset). Broad terms that contain more than 30 genes were excluded from further analysis, as well as terms with less than 3 genes.

### 2.7 GO enrichment analysis

For the GO enrichment analysis, we used the PANTHER web API (http://pantherdb.org/services/openAPISpec.jsp) with annotation files from March 2022 (GO Ontology database DOI:10.5281/zenodo.6399963 released on 2022-03-22). We employed the ‘Enrichment (Overrepresentation)’ test which computes a *p* − *value* using Fisher’s exact test, and the False Discovery Rate (FDR) method was used for multiple hypothesis correction. We limit the enrichment testing to only include biological process terms. In order to avoid broad terms, we restricted the analysis to terms that contain at most 250 genes. The test cutoff was set to an FDR *q* − *value* < 0.05.

### 2.8 Feature generation

We designed four sets of features that span a variety of molecular functions including genetic interactions, pathway maps, biological processes, and protein complexes. We refer to a feature based on the main dataset it was derived from, namely, KEGG, GO BP, CYC2008, and GO CC. A summary of all the feature sets we evaluated for telomere length classification is presented in Table 1.

**TABLE 1:** Overview of the feature sets used to evaluate *S. cerevisiae* telomere length classification pipelines.

	Number of samples			Number of features
Feature name	Short TLM	Long TLM	Total	
GO BP	264	166	430	1559
KEGG	264	166	430	109
CYC2008	169	88	257	146
GO CC	166	78	244	200
GO BP/KEGG	264	166	430	1668
GO BP/CYC2008	152	82	234	1705
GO BP/GO CC	166	78	244	1759
KEGG/CYC2008	152	82	234	255
KEGG/GO CC	166	78	244	309
CYC2008/GO CC	129	58	187	346

In order to extract the KEGG and GO BP features, we considered for each gene its proportion from the group that has a non-zero GI score with it. For KEGG and GO BP, respectively, this group consists of the genes that make up a pathway and GO term direct gene members, more details are provided in the Supplementary Material. The CYC2008 and GO CC features indicate for each protein complex a gene’s membership in it. When combining a pair of feature sets, it was done by merging on the intersection of genes that are in both sets.

For classifying TLM genes and predicting *S. pombe* candidates, we defined another feature, namely, propagation to anchor genes. These are genes that act as an endpoint for TLM-related processes as described for *S. cerevisiae* (Shachar et al., 2008) and that we adapted for *S. pombe* (Supplementary Table S3). Producing this feature employs a random walk with a restart propagation process as described in (Cowen et al., 2017). Specifically, the following steps were taken:

1) We used the whole genome with Protein-Protein Interaction (PPI) binary scores from BioGRID v4.4.207 as the adjacency matrix (interactions labeled ‘physical’ in their ‘Experimental System Type’ field). We normalized this matrix by *W* = *AD* ^−1^, where *A* is the adjacency matrix and *D* is the diagonal degree matrix.
2) The starting vector *p*_0_ was set to $\frac{1}{\left|G\right|}$ for each anchor gene and zero for all other genes, where |*G*| is the number of anchor genes.
3) The resulting feature vector was produced by a computation until convergence of the vector *p*_*k*_ = 0.2*p*_0_ + 0.8*Wp*_*k*−1_

### 2.9 Machine learning models

To evaluate classification performance, we used five standard machine learning models from the Python package SciKit-Learn (version 1.0.2), that have been proposed for obtaining good prediction accuracy in the bioinformatics domain (Olson et al., 2018). We retained the recommended hyperparameters that were set prior to all experimentations as summarized in Table 2.

**TABLE 2:** Machine learning models used, and their parameters. Parameters that are not in the table were set to the default values of the SciKit-Learn (version 1.0.2) package.

Model name	Parameters
XGBClassifier (XGB)	n_estimators = 500
	max_depth = 3
	max_features = 'log2′
	eval_metric = 'logloss’
	learning_rate = 0.1
RandomForestClassifier (RF)	n_estimators = 500
	criterion = 'entropy’
	max_features = 0.25
ExtraTreesClassifier (ET)	n_estimators = 1000
	max_features = 'log2′
	criterion = 'entropy’
SVC (PSVC)	C = 0.01
	gamma = 0.1
	degree = 3
	coef0 = 10
	kernel = 'poly’
LinearSVC (LSVC)	max_iter = 100000
LogisticRegressionCV (LRCV)	max_iter = 10000

This list of models was tweaked to include the Extreme Gradient Boosting Classifier (XGB) from the XGBoost package (v0.9) instead of the Gradient Boosting Classifier and the Logistic Regression was replaced by the Logistic Regression Cross Validation (LRCV) model. In addition, the Linear Support Vector Classifier (LSVC) model was added to have more than one linear model assessed, resulting in six models overall.

### 2.10 Evaluation setting

Our dataset is imbalanced among the telomere length classes. This leads to models that are overly conservative when predicting the minority classes. To address this issue we performed 5 repeated stratified 10-fold Cross-Validation (CV) experiments. This procedure is followed for each held-out test in a 10-fold CV and the whole process is repeated 5 times, producing different splits and held-out test sets in each repetition, but maintaining the percentage of samples for each class. For all the setups, the models were assessed in each run for the relevant evaluation metrics on the held-out test dataset and the median score (unless stated otherwise) across all experiments is reported.

The evaluation metrics included Matthew’s Correlation Coefficient (MCC) and Area Under the receiver-operating characteristic Curve (AUC) as they are more robust to imbalanced label distribution (He and Garcia, 2009; Chicco and Jurman, 2020). The MCC is calculated as follows:

$$MCC=\frac{TP\times TN-FP\times FN}{\sqrt{\left(TP+FN\right)\left(TP+FP\right)\left(TN+FP\right)\left(TN+FN\right)}}$$ (1)

where TP represents the True Positive; TN, the True Negative; FP, the False Positive; FN, the False Negative. It is the Pearson correlation coefficient between the predicted and true labels. The Receiver-Operating Characteristic curve depicts the true positive rate as a function of the false positive rate. The AUC is a metric for assessing a classifier’s overall performance. The better the classifier is, the closer to one the AUC is.

In order to keep the number of features not greater than the number of samples (Ressom et al., 2008), feature sets that exceeded this threshold were reduced to match the size of the samples in all of the experiments. To this end, a Bernoulli Naive Bayes Classifier was applied during the training phase to determine the importance of each feature based on the observed log probability of features given a class. The top ranking features, up to the number of samples, were selected to be used for testing *via* SciKit-Learn’s SelectFromModel.

## 3 Results

### 3.1 Prediction of telomere length changes following gene knockout

We compiled a comprehensive collection of feature sets for telomere length prediction following gene knockout. First, we focused on the task of predicting shorter-than-normal vs. longer-than-normal length. We assessed each one of the feature sets individually after applying standardization scaling using the StandardScaler functionality from SciKit-Learn.

**FIGURE 1:**
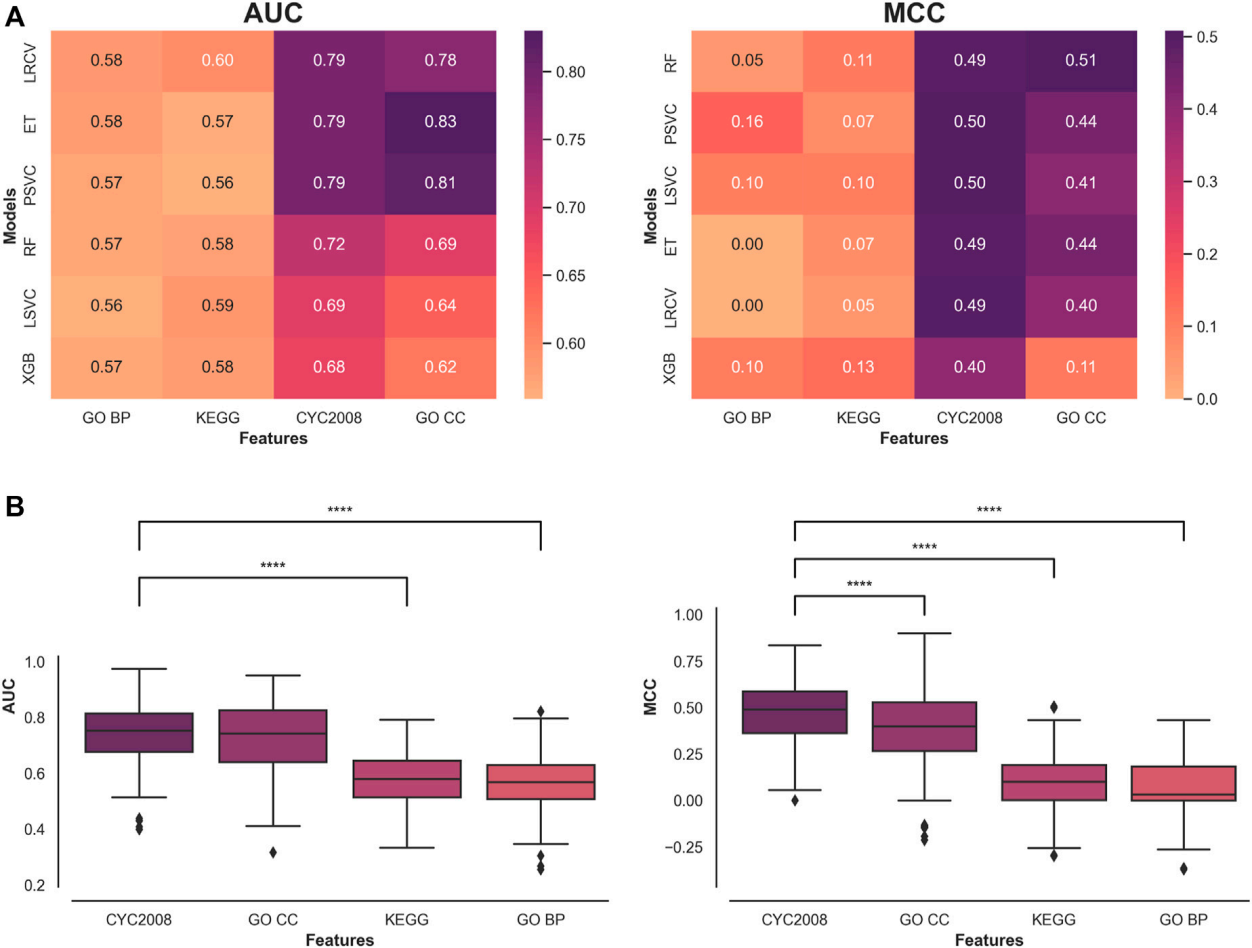
Comparative assessment of feature set performance in classifying short and long phenotypes. **(A)** Median model performance for each feature set. **(B)** Performance distribution of the features with a Mann-Whitney U test with continuity correction comparing the top feature set with all the rest with the significant comparisons indicated. **** indicates *p* < 0.0001.

A heatmap of the metrics’ median results across all test runs for each Machine Learning (ML) model and feature sets is presented (Figure 1A). The results are ordered by the overall median score of a model, across all features. The Random Forest Classifier (RF) achieved the greatest performance across all features in the MCC metric with an overall median score of 0.3 and LRCV had the highest overall median AUC score of 0.69.

A comparison of the type of features used reveals that protein complex-based features (CYC2008 and GO CC) significantly outperform other features across all classification metrics and ML models in this context (Figure 1B). Comparing the two in each metric shows that CYC2008 has a significantly higher MCC score (0.49 vs. 0.39, *p* < 0.0001), but achieves similar AUC scores (0.75 vs. 0.74). Therefore, ML models using a single set of protein complex-based features, and in particular, CYC2008, will lead to a better classification of telomere length samples than pathway features.

**FIGURE 2:**
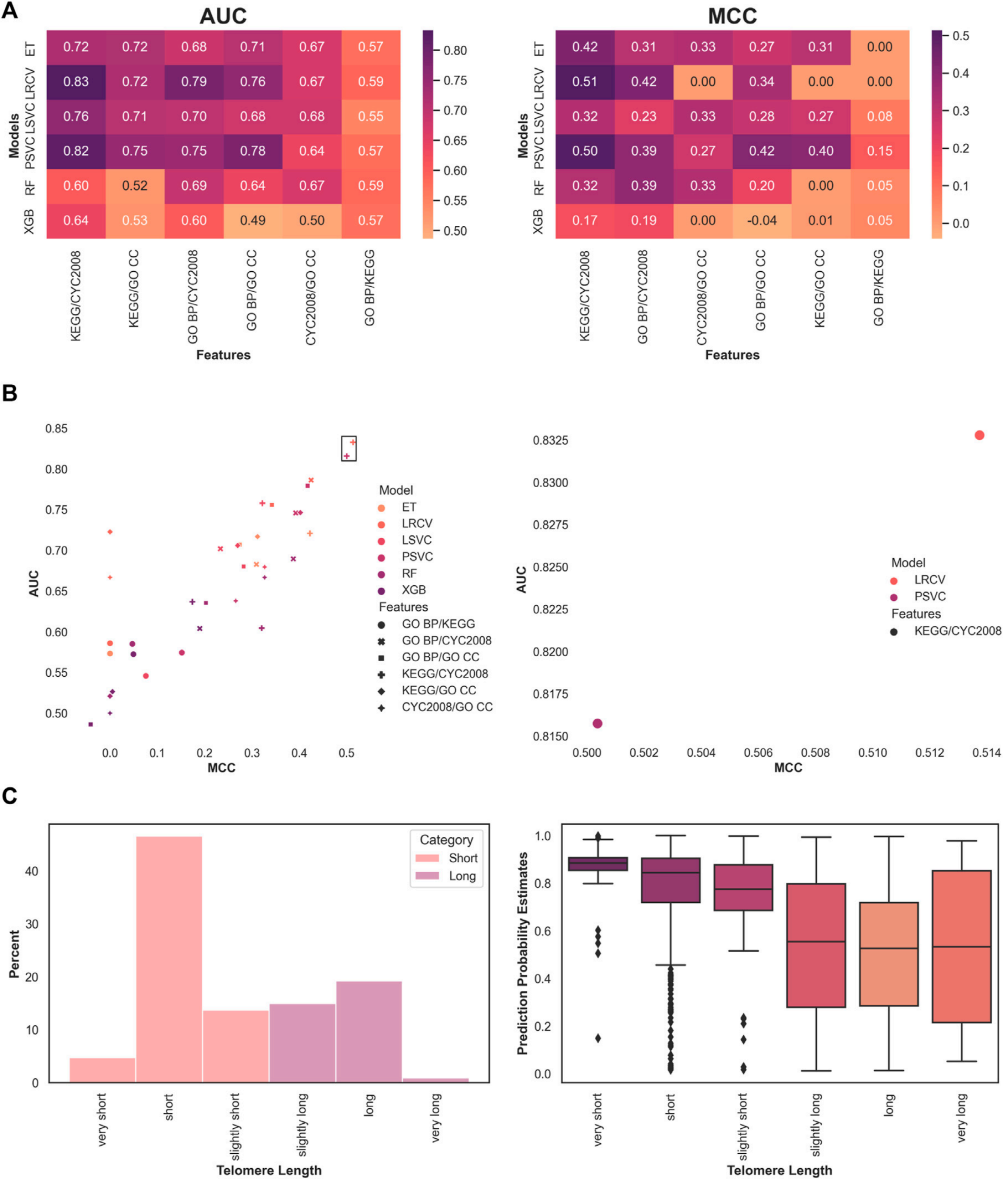
Optimal classification of short and long telomere lengths is obtained with pathway and complex-based features using a linear model. **(A)** Median model performance for each pairwise feature set. **(B)** Overall best performing model across features. The top two are marked by a frame and enlarged. **(C)** Showing the phenotype distribution of the 1170 samples analyzed for various lengths in *S. cerevisiae* using our system, and the probability estimates for each label across all experiments.

Next, we moved to examine a mixture of feature sets. We limited the search to pairwise feature space combinations, selecting the top features in the same manner as described above. The results are ordered by the median score per feature combination, across all models (Figure 2A). In both AUC and MCC measures, the highest-performing features across all models are made of the combination of protein complex features (CYC2008) and pathway features (KEGG). The highest overall performing model and feature set were the LRCV with CYC2008 and KEGG features, with a median AUC score of 0.834 and median MCC score of 0.513 across all experiments (Figure 2B). In addition, the top two performing models in both metrics used the CYC2008 and KEGG feature combination, both exceeding the median AUC > 0.8 and MCC > 0.5 scores, attesting to the utility of these features. Therefore, our subsequent analysis utilized only CYC2008 and KEGG feature sets with the LRCV model pipeline.

Finally, we performed a meta-analysis of the predictions. Instead of the binary categorization to ‘short’ and ‘long’ phenotypes, we checked to see if the classifier’s estimated probabilities for those predictions were higher in more extreme phenotypes. To this end, we looked at the original phenotypes, namely, ‘very short’, ‘short’, ‘slightly short’, ‘slightly long’, ‘long’, and ‘very long’. They were originally (Askree et al., 2004) deemed as such by comparing them to a baseline of wild-type telomere length, measuring in bulk Southern blot. For example, strains of 385–420 telomeric nucleotides were considered ‘long’ when compared to the 350±35 nucleotides of the wild-type, whereas those with longer telomeres were designated ‘very long’. We compared the estimated probabilities that were produced by the model for each such subtype (Figure 2C). When focusing on the larger fraction (about 67%) of phenotypes that were reduced to ‘short’, we observed that severe phenotypes were assigned with higher confidence (median probability of 'very short’ 0.88 vs. 'short’ 0.84, *p* < 0.0005, and 'short’ vs. 'slightly short’ 0.77, *p* < 0.0005). This was not the case for the ‘long’ phenotypic length (no significant differences were found between ‘slightly long’, ‘long’, and ‘very long’). One plausible explanation is the relatively small number of samples that were available during training and testing (for example, less than 1% is labeled ‘very long’).

### 3.2 Application to *Schizosaccharomyces pombe*

**FIGURE 3:**
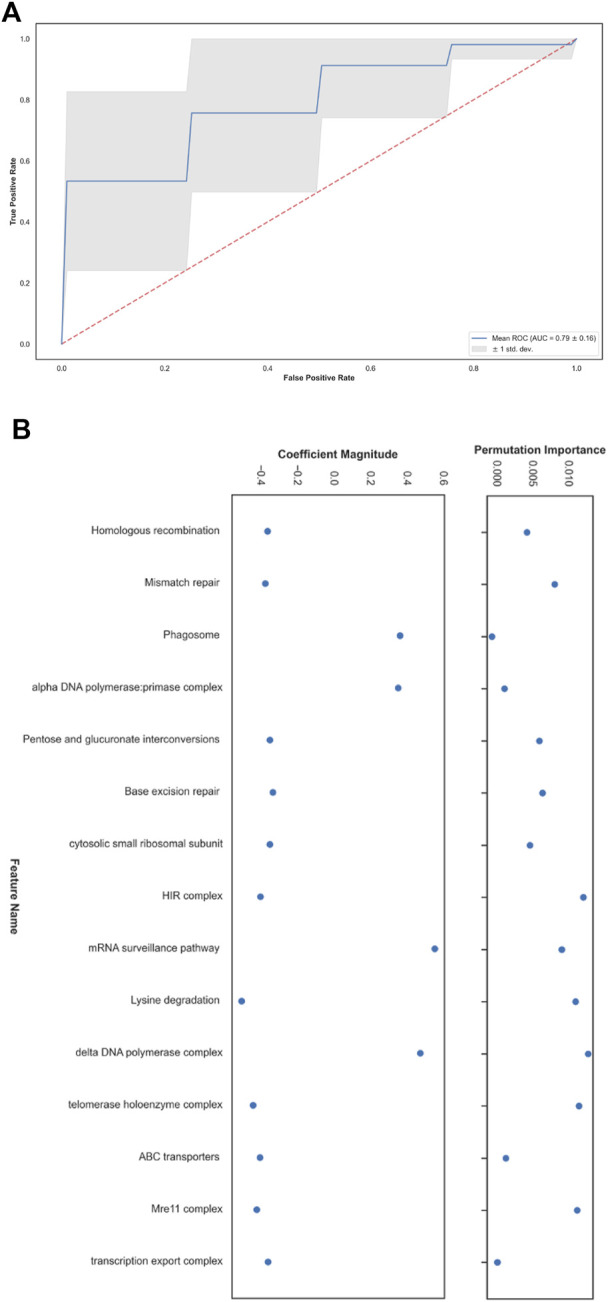
Short and long telomere length prediction in *Schizosaccharomyces pombe*. **(A)** Receiver operating characteristic (ROC) plot. **(B)** The top fifteen features with the greatest contribution to the classification according to coefficients learned by the linear model and their permutation importance.

After establishing our classifier’s performance, we wished to generalize it to data from *S. pombe*, a fungal species whose ancestors separated from *S. cerevisiae* ≈ 400 million years ago (thus these species are different from each other as either is from animals). To this end, we first mapped *cerevisiae* features to *pombe* features. KEGG pathways contain a consistent naming convention, allowing for these features to be mapped seamlessly. For the complex-based features, we retained the ones in CYC2008 that have a unique GO identifier and intersected that set with the *S. pombe* corresponding set (with the same GO ID). Overall, we could reproduce 127 features for 198 samples (‘long’ - 158, ‘short’ - 40). As before, we trained a ‘short’/‘long’ classifier and evaluated its performance in cross-validation. The median AUC score for classifying short and long telomere length was 0.81, mean AUC result of 0.79 with a standard deviation of 0.16 (Figure 3A) and a median MCC of 0.49.

Next, we turned to analyze the feature importance that led to these results (Figure 3B). To accomplish this, we took the fifteen coefficients with the highest mean absolute value that the LRCV model learned across all runs. Using the held-out set in each run, allowed us to detect which characteristics contribute the most to the examined model’s generalization capability by applying the SciKit-Learn’s permutation_importance with n_repeats = 5. The difference between the baseline scoring metric that is used by LRCV (the default accuracy measure, in our case) and the one from permuting the feature column is defined as the permutation importance. To summarize, after running all the experiments, we were left with the top-15 highest mean absolute coefficients that participated in all the runs and their respective mean permutation importance. Having our data scaled in the preprocessing step, allowed us to then look at the odds ratio for each feature in conjunction with its permutation importance score with regards to rest. This way, we could assess the highest influencing features in our system as-is, disregarding interacting terms. Among the features with a relative high permutation score, we find the telomerase, *Mre11-Rad50-Xrs2* (MRX) and the HIstone Regulator (HIR) complexes and DNA damage response pathways, aligning with the findings of the *S. cerevisiae* TLM mechanisms (Askree et al., 2004; Rubinstein et al., 2014). Despite the difference between the data sets of the two organisms where in *cerevisiae* the majority group is the ‘short’ one, while in *pombe* the opposite is true, the performance generalized well to the *pombe* setting.

### 3.3 Prediction of *S. pombe* TLM genes from orthologs

Orthologs are the result of speciation events and are likely to be functionally related. In our context, previous research has demonstrated that gene dispensability is conserved for the majority of ortholog genes in budding and fission yeast (Kim et al., 2010). Based on this result, we set to assess if the same holds for TLM genes. According to data we have, out of 3953 orthologs, only 51 genes (1.29%) are TLM genes in both species. However, when focusing on the subset of TLM genes in either species, 9.94% of the genes (51/513) are TLM genes in both yeasts. A closer look into the telomere length phenotype within this subset of shared TLM orthologs demonstrates more robust conservation than the one that has been detailed so far. 60.78% (31/51) of shared TLM genes preserve the phenotype (*p* < 0.1). Overall, 29.4% (15/51) and 31.37% (16/51) of these orthologous pairs retain the TLM ‘short’ and ‘long’ phenotypes, respectively.

We postulate that there could be more *S. pombe* TLM genes within the orthologous pairs that are currently unknown to have this role. In order to predict TLM candidates, our prediction system was evaluated against the task of classifying between TLM and non-TLM genes. To this end, the LRCV model remained the same, apart from the additional setting of its class_weight parameter to 'balanced’. The KEGG and CYC2008 feature sets were built in the same manner, but this time with respect to the entire ortholog gene set. In total, we explored five methods for TLM prediction (as also summarized in Table 3):

• Method (1): Predicting a role (TLM/non-TLM) for an *S. pombe* gene based on its *S. cerevisiae* ortholog.
• Method (2): Training a predictor on *S. cerevisiae* data and applying it to *S. pombe* data.
• Method (3): Training and testing on *S. pombe* data.
• Method (4): Similar to (3), but utilizing also the *S. cerevisiae* features of the gene’s ortholog.
• Method (5): Similar to (3), but with the addition of a feature quantifying the proximity of the gene to anchor TLM genes.

**TABLE 3:** Overview of datasets used in different methods for classifying TLM and non TLM genes.

Method	Species	TLM	Non TLM	Total	Method	Feature sets	Number of features
	*S. pombe*	158	2445	2603	1	Ortholog	1
1–4	*S. cerevisiae*	254	2068	2322	2–3	KEGG, CYC2008	259
					4		518
5	*S. pombe*	134	1884	2018	5	KEGG, CYC2008, Anchor genes	260

The performance of the five predictors is summarized in Table 4. Method (5) performed best; out of the other four methods, method (3) – applying our framework as-is to this new task – dominated the rest in terms of AUC.

**TABLE 4:** Evaluation of methods used in classifying TLM and non TLM genes. Rows are sorted in ascending order by AUC performance. The highest scores in each metric are marked in bold.

Method	AUC	Recall	Precision
1	0.601	0.297	0.169
2	0.628	0.151	0.137
4	0.686	0.387	0.202
3	0.71	0.387	0.186
5	**0.771**	**0.461**	**0.236**

Next, we aimed to predict new TLM candidates in *S. pombe* using method (5). To this end, we executed the model in a leave-one-out setting so that we could use the entire dataset for training and make a prediction with respect to each gene in turn. The resulting top-30 predictions that are not known to be TLM genes, along with their *S. cerevisiae* orthologs, were subjected to GO enrichment analysis (Supplementary Tables S4–S6). The top-10 is presented in their ranked order (Table 5). Homing in on some of the genes shows that they are associated with the Target of Rapamycin (TOR) signaling network (Ungar et al., 2011; Rallis et al., 2017; Lie et al., 2018) a known participant in the regulation of subtelomeric and telomeric regions (Schonbrun et al., 2009; Cohen et al., 2018). Further inspection reveals that 10% of the *S. pombe* predicted genes are orthologous to known TLM genes in *S. cerevisiae*. This is reassuring as it is consistent with the prior experimental knowledge that was discussed above. Overall, our candidate TLM genes were significantly enriched (*p* < 0.001) for core DNA maintenance processes (DNA damage response, DNA repair, and DNA replication), DNA assembly or remodeling functions (chromatin organization, chromosome segregation), and mitotic and meiotic cell cycles (regulation of cell cycle process, regulation of mitotic cell cycle and meiotic cell cycle). Taken together, these enriched terms suggest that there is an association between our candidate genes and telomere maintenance homeostasis.

**TABLE 5:** The top-10 *S. pombe* TLM predicted genes and their *S. cerevisiae* orthologs. The rows are sorted in descending order by the model’s estimated probability of the prediction.

Systematic name *S. pombe*	Gene name *S. pombe*	Product description	Systematic name of *S. cerevisiae* ortholog	Gene name of *S. cerevisiae* ortholog
SPCC 1919.03c	*amk2*	serine/threonine protein kinase AMPK (beta) regulatory subunit Amk2	YGL208W	*SIP2*
SPCC31H12.08c	*ccr4*	CCR4-Not complex	YAL021C	*CCR4*
		3′-5′-exoribonuclease		
		subunit 6		
SPCC1259.13	*chk1*	Chk1 protein kinase	YBR274W	*CHK1*
SPBC947.08c	*hip4*	histone H3.3-H4	YBR215W	*HPC2*
		chaperone, HIR complex		
		subunit Hip4		
SPBC725.16	*res1*	MBF transcription factor	YER111C	*SWI4*
		complex subunit Res1		
SPAC25A8.01c	*fft3*	SMARCAD1 family	YAL019W	*FUN30*
		ATPase Fft3		
SPCC18B5.11c	*cds1*	DNA replication	YPL153C	*RAD53* (*)
		checkpoint kinase Cds1		
SPAC4G8.13c	*prz1*	DNA-binding transcription	YNL027W	*CRZ1*
		factor, calcineurin		
		responsive Prz1		
SPAC607.09c	*btn1*	battenin CLN3	YJL059W	*YHC3*
		family protein		
SPAC1687.15	*gsk3*	serine/threonine	YMR139W	*RIM11*
		protein kinase Gsk3		

(*) indicates a known budding yeast TLM gene. Product description data was taken from PomBase (Harris et al., 2022).

## 4 Conclusion

This study set out to create a general machine learning pipeline for telomere length maintenance analysis in fission and budding yeast. We have identified a set of features along with a simple linear model that can predict the telomere length phenotype under various settings. The framework can also pinpoint explanatory variables leading to its output while utilizing a broad range of data sources, including genetic interaction data, that is being used for the first time in this context, to the best of our knowledge.

The generalizability of these results is subject to certain limitations. For instance, the datasets used to derive the features are incomplete and so is our interpretation of the predictions. Furthermore, the small and imbalanced data, in some of the tasks we investigated, makes it hard to learn the underlying structure of the data.

Although this study focuses on yeast datasets, the suggested system may well have a bearing on human data, such as the UK Biobank (Bycroft et al., 2018) as telomere length is a promising biomarker for age-associated diseases and cancer. Considerably more work will need to be done in order to have high quality data of the *S. pombe* TLM network, and our predictions are a promising starting point for this investigation.

## Data Availability

The genetic interaction data used for *S. cerevisiae* contains no version control and was downloaded from https://thecellmap.org/costanzo2016/ on March 2022. For the rest of this work, publicly accessible datasets were examined, and the publication makes note of the relevant versions. All of the data and code used for this study are available at: https://github.com/Iftahp/yeastTLM.
