# Supplementary material for: A comparative analysis of telomere length maintenance circuits in fission and budding yeast
Source: Front Genet. 2022 Nov 4;13:1033113. doi: 10.3389/fgene.2022.1033113 (PMC9672475; doi:10.3389/fgene.2022.1033113)
Supplement: Supplementary file 1 [file DataSheet1.PDF]

Supplementary Material

1 SUPPLEMENTARY INFORMATION

1.1 Feature generation

Here we give further clarification of the process of extracting the KEGG and GO BP features. Formally, define the GI score matrix as $X \in \mathbb{R}^{n \times q}$, where n is the number of unique genes and q are the genes that interact with at least one of those genes. Each entry, $x_{i,j}$, represents the GI score between gene i and j. If X contains decimal values, it was converted to a binary matrix $K \in \mathbb{Z}_2^{n \times q}$ using the following transformation:

$$k_{i,j} = \begin{cases} 1 & \text{if } x_{i,j} \neq 0 \\ 0 & \text{if } x_{i,j} = 0 \end{cases} \tag{S1}$$

For the final step, K was mapped to a feature per gene and pathway, such that for every pair (g, p) of a gene g and a pathway p, we measure the fraction of GIs between g and the gene members of p as:

$$Feature(g, p) = \frac{\sum_{i \in p} k_{i,g}}{|p|} \tag{S2}$$

where $||$ denotes the cardinality operator.

The process for generating GO BP is identical, but instead of using pathways that contain genes, terms of GO biological process with their direct gene members were used.

2 SUPPLEMENTARY TABLES AND FIGURES

All of the **Supplementary Tables** were uploaded in a single Zip file. These files could also be reproduced using the GitHub repository code.
The following are the matching captions for each **Supplementary Table**:

- **Table S1**. TLM genes with corresponding binary telomere length phenotype for *S. cerevisiae* and *S. pombe*. The column labeled ”Original Phenotype” of *S. cerevisiae* contains information on the phenotype before reduction.
- **Table S2**. *S. pombe* to *S. cerevisiae* orthologous pairs. For each *S. pombe* gene there is a single corresponding *S. cerevisiae* ortholog.
- **Table S3**. Genes of *S. pombe* used in the feature ’propagation to anchor genes’. The *S. cerevisiae* genes from Shachar et al. (2008) are given for reference.
- **Table S4**. Top-30 *S. pombe* TLM candidates and their *S. cerevisiae* orthologs.
- **Table S5**. GO enrichment analysis of the top-30 *S. cerevisiae* orthologs of the TLM candidates.
- **Table S6**. GO enrichment analysis of the top-30 *S. pombe* TLM candidates.

REFERENCES

Shachar, R., Ungar, L., Kupiec, M., Ruppin, E., and Sharan, R. (2008). A systems-level approach to mapping the telomere length maintenance gene circuitry. *Molecular systems biology* 4, 172
