# Supplementary material for: A comparative analysis of telomere length maintenance circuits in fission and budding yeast
Source: Front Genet. 2022 Nov 4;13:1033113. doi: 10.3389/fgene.2022.1033113 (PMC9672475; doi:10.3389/fgene.2022.1033113)
Supplement: Supplementary file 2 [file DataSheet2.ZIP › Supplementary material/Table S3 anchor genes.pdf]

Species	Standard Name	Systematic Name	Name Description/ Product
S. cerevisiae	EST1	YLR233C	Ever Shorter Telomeres
	EST2	YLR318W	Ever Shorter Telomeres
	EST3	YIL009C-A	Ever Shorter Telomeres
	YKU70	YMR284W	Yeast KU protein
	YKU80	YMR106C	Yeast KU protein
	STN1	YDR082W	Suppressor of cdc ThirteeN
	TEN1	YLR010C	TElomeric pathways with STn1
	CDC13	YDL220C	Cell Division Cycle
	EXO1	YOR033C	EXOnuclease
	RAP1	YNL216W	Repressor/Activator site binding Protein
S. pombe	taz1	SPAC16A10.07c	shelterin complex subunit Taz1
	rap1	SPBC1778.02	shelterin complex telomere binding subunit Rap1
	poz1	SPAC19G12.13c	shelterin complex subunit Poz1
	tpz1	SPAC6F6.16c	shelterin complex subunit Tpz1
	pot1	SPAC26H5.06	shelterin complex subunit Pot1
	ccq1	SPCC188.07	shelterin complex HEAT repeat subunit Ccq1
	stn1	SPBC409.12c	telomere cap complex subunit Stn1
	ten1	SPCC1393.14	nuclear telomere cap complex subunit Ten1
	est1	SPBC2D10.13	telomerase regulator Est1
	trt1	SPBC29A3.14c	telomerase reverse transcriptase 1 protein Trt1
	pku70	SPCC126.02c	Ku domain helicase (human XRCC6 ortholog) Pku70
	pku80	SPBC543.03c	Ku domain helicase (human XRCC5 ortholog) Pku80

Supplementary Table S3 | *S. pombe* Genes used in the feature ‘propagation to anchor genes’. The *S. cerevisiae* genes from (Shachar et al. 2008) are given for reference.
